# The Impact of Previous Exposure to COVID-19 on the Outcome of ICSI Cycles

**DOI:** 10.5935/1518-0557.20220063

**Published:** 2023

**Authors:** Kamal Eldin Abdullah Rageh, Elsayed Ali Farag, Mohamed Atef Behery, Mohamed Ahmed Badreldin, Eman Ahmed Ali

**Affiliations:** 1 Lecturer of Obstetrics and Gynecology, Faculty of Medicine, Al-Azhar University, Cairo, Egypt; 2 IVF Consultant, Albaraka Fertility Hospital, Manama, Bahrain; 3 Assistant Professor of Obstetrics and Gynecology, Faculty of Medicine, Al-Azhar University, Cairo, Egypt; 4 Consultant of Obstetrics and Gynecology, Almana General Hospital, IVF unit, Western Provenance, Saudi Arabia; 5 Assistant Professor of Obstetrics and Gynecology, Assisted Reproduction Unit, Al-Azhar University, Cairo, Egypt; 6 Consultant of Obstetrics and Gynecology, Assisted Reproduction Unit, Al-Azhar University, Cairo, Egypt

**Keywords:** COVID-19, infection, ICSI, reproduction, outcome

## Abstract

**Objective:**

Due to the large increase in the number of reported cases and the impact of COVID-19 on public health, the European Society for Human Reproduction and Embryology (ESHRE) recommended the cessation of all activities related to assisted reproduction. There are many unknowns about the long-term effects of the virus on fertility and pregnancy. We conducted this study to offer some evidence-based guidance on the relationship between COVID-19 and IVF/ICSI cycle outcomes.

**Methods:**

This observational study included 179 patients who underwent ICSI cycles at the Albaraka Fertility Hospital, Manama, Bahrain and the Almana hospital, KSA. The patients were divided into two groups. Group 1 included 88 individuals with a history of Covid-19 and Group 2 included 91 subjects without a history of COVID-19.

**Results:**

Despite the higher pregnancy (45.1% *vs*. 36.4%, with *p*=0.264) and fertilization (52% *vs*. 50.6% with *p*=0.647) rates seen in patients without a history of COVID-19, the differences were not statistically significant.

**Conclusions:**

There is no clear evidence that exposure to COVID-19 significantly affects ICSI cycle outcomes.

## INTRODUCTION

Since the first case identified in Wuhan (China) in late December 2019, severe acute respiratory syndrome coronavirus 2 (SARS-CoV-2) infection, or COVID-19 has developed into a global pandemic. As of 24 November 2021, more than 258 million cases have been confirmed, with more than 5.16 million deaths attributed to COVID-19, making it one of the deadliest pandemics in history (WHO, 2021a). The World Health Organization (WHO) declared a Public Health Emergency of International Concern on January 30, 2020 (WHO, 2020a). The pandemic was declared on March 11, 2020 (WHO, 2020b). The severity of COVID-19 symptoms is highly variable, ranging from un-noticeable to life-threatening. Severe illness is more likely in the elderly and individuals with underlying medical conditions.

The diagnosis of COVID-19 can be made based on symptoms and known exposure or simply from a positive test for SARS-CoV-2 even in the absence of any symptoms. COVID-19 can, therefore, be symptomatic or asymptomatic. Mortality occurs mainly from severe lung involvement causing acute respiratory distress syndrome, although sometimes multi-organ failure occurs, with significant coagulation disorders (Zhou *et al*., 2020). The World Health Organization publishes a weekly international status report with an additional Situation Dashboard to provide information for individual countries.

Due to the large increase in the number of reported cases and the impact of COVID-19 on public health, the European Society for Human Reproduction and Embryology (ESHRE) on March 19, 2020, and the American Society for Reproductive Medicine (ASRM) on March 30, 2020, recommended the cessation of all activities related to assisted reproduction. Then, both societies, ESHRE on April 23 and ASRM on April 24, authorized the resumption of healthcare activities with the general safety recommendations established by government authorities in each country (Veiga *et al.,* 2020). These measures aimed at minimizing contagion and were also based on the fact that infertility is a disease whose prognosis can worsen over time. Protocols have been established to minimize the risk of contagion for both patients and staff. However, few measures have been established regarding ovarian stimulation protocols, triggering, and other considerations related to the clinical management of patients (Veiga *et al.,* 2020).

Whilst the global COVID-19 pandemic is still evolving and there are many unknowns about the long-term effects of the virus on fertility and pregnancy, we found ourselves in need to offer some evidence-based guidance. This is why we decided to carry out this study, in which we evaluated the relationship between COVID-19 and IVF/ICSI cycle outcomes. Before we dive into some of the preliminary research, it is worth underscoring that COVID-19 is relatively new and data is still not set in stone. What this means is that we only have two years’ worth of data to study. Although some clues may be derived from the data available, we are still far from fully understanding the short- and long-term effects of COVID-19. To complicate matters, the virus appears to be mutating, with new strains coming out of the United Kingdom, South Africa, Brazil, and India so far.

As you read on, please bear this in mind, and rest assured that we are following the science vigilantly so we can pass on any important information to our patients.

## MATERIALS AND METHODS

### Study design

This observational study included 179 patients who underwent ICSI cycles at the Albaraka Fertility Hospital, Manama, Bahrain and Almana hospital, KSA between August 2020 and May 2021. The local ethics committees approved the study and granted it certificate number NCT05198401. We compared the outcomes of patients with a history of COVID-19 (study group, n=88) with the outcomes of non-infected comparable patients (control group, n=91).

### Study population

- **Inclusion criteria:** The study included women who recovered from Covid-19 and had been without disease for at least three months, based on their history with positive test results confirmed via the BeAware Bahrain phone application and the Tawakkalna Saudi phone application. They were aged less than 38 years, had a body mass index (BMI) below 30 kg/m^2^, and were offered the antagonist protocol for IVF.
- **Exclusion criteria:** Individuals with chromosomal and genetic disorders, with ages > 38 years, BMI > 30, abnormal ultrasonogram of uterine cavity (acquired or congenital anomalies), patients given the long agonist protocol, cases with severe male factor, and with abnormal embryos not suitable for transfer were excluded. We also excluded patients vaccinated against COVID-19 and individuals with current infection.

### Study protocol

Before the start of treatment: A screening questionnaire was completed. Patients were evaluated clinically with consideration to antigen PCR testing to exclude current COVID-19 infection. Then, before each course of treatment, patients were evaluated using baseline serum hormone tests for FSH, luteinizing hormone (LH), and estradiol (E2). Baseline transvaginal ultrasound scanning was performed to assess AFC and to rule out the presence of ovarian cysts. To calculate total AFC, the number of follicles measuring 2 to 9 mm in both ovaries was evaluated. All sonographic examinations were performed with a 7.5mHz endovaginal probe of a SIEMENS, ACUSON NX2 ultrasound device. During treatment: A coronavirus screening questionnaire was administered prior to every visit to the clinic. Only patients who remained negative on questionnaire screening were allowed to complete treatment.

### Stimulation protocol

The included patients underwent individualized antagonist protocol; Gonadotropin treatment was initiated on the 2^nd^ or 3^rd^ day of the cycle if no follicles were larger than 10 mm in diameter. The starting dose of gonadotropin of recombinant FSH GONAL-f (Merck Serono) was based on age, AMH, AFC, and BMI. Ovarian response to COS was monitored by transvaginal US and serum estradiol (E2) levels from Day 6 of stimulation. Once the leading follicle reached a size of 13 mm, co-treatment with GnRH antagonist 0.25 mg/day (Cetrotide, Serono) and highly purified human menopausal gonadotropin (Menopur, Ferring, Toronto, Ontario, Canada) were commenced. The dose of gonadotropin was adjusted as per individual requirement. Follicle growth and hormone levels were serially monitored by ultrasound and estradiol (E2) level. When the dominant follicles reached an average diameter of 18–20 mm, patients were triggered for final follicular maturation with hCG (Ovitrelle 250 micrograms/0.5 ml prefilled pen; Merck) and oocyte retrieval was performed 34-36 hours later under sedation. Intracytoplasmic sperm injection was performed in all the patients. Oocyte maturity and embryo grading were done as per the laboratory protocol, and then embryo transfer was performed on cleavage-stage Day 5 using a soft catheter (Wallace). The number of embryos transferred was determined by the available number and quality of embryos and by the guidelines of the institution and the ASRM Practice Committee. All patients were given supplementation with natural micronized progesterone (Endometrin 100, Ferring, USA) given vaginally twice daily, and oral dydrogesterone 10 mg twice daily (Duphastone, Abott) beginning on the day of oocyte retrieval. The patients received supplementation with oral 6-mg doses of micronized 17 b-estradiol (Estrofem; Novo Nordisk, Denmark) daily during the entire luteal phase.

### Outcomes

The primary outcome was clinical pregnancy. Secondary outcomes included the number of mature oocytes (MII), fertilization rate, and grade 1-2 embryos.

### Statistical analysis

Microsoft Excel 360 software for Windows was used for data collection, validation and processing. SPSS version 22 (IBM, New York, USA) was used for data analysis.

Normality tests (Kolmogrov-Semrinov and Shapiro-Wilk) revealed the data followed a normal distribution. Therefore, parametric tests were used for comparison.

The following tests were performed:

**Student’s t-test:** A parametric test used to compare the outcomes between two independent groups, the means between two populations, and quantitative variables.

**Chi-squared test:** A Chi-squared (χ^2^) is a measure of the difference between the observed and expected frequencies of the outcomes of a set of events. It is used to compare between categorical variables.

**Probability (*p*-value)**

- *p*-values < 0.05 were considered significant.

## RESULTS

Two groups were compared. Group 1 (n=88) included patients with a history of COVID-19 and Group 2 (n=91) included patients without a history of COVID-19. Comparison of baseline characteristics between groups found no significant differences regarding age, (32.14±4.773 *vs.* 31.42±4.971; *p*=0.325), BMI (26.2649±3.17425 *vs.* 26.4531±3.51008; *p*=0.707), infertility duration (5.614±3.1291 *vs.* 6.374±3.7324; *p*=0.142), baseline FSH level (6.394±1.3778 *vs.* 6.043±1.5478; *p*=0.111), antral follicle count (AFC) (10.65±3.514 *vs.* 9.95±3.874; *p*=0.206), duration of stimulation (10.32±1.255 *vs.* 10.13±1.368; *p*=0.344), number of oocytes retrieved (10.93±6.739 *vs.* 11.89±7.484; *p*=0.370), number of MII oocytes (7.9±4.8 *vs.* 8.9±5.8; *p*=0.0233), number of embryos transferred (2.13±.907 *vs.* 2.24±.947; *p*=0.401), number of grade 1-2 embryos (1.51±1.322 *vs.* 1.43±1.309; *p*=0.674) and day of embryo transfer (3.59±.997 *vs.* 3.76±1.013; *p*=0.301) in Groups 1 and 2, respectively (Table 1).

**Table 1:** Baseline characteristics and outcomes of both groups.

Variables	Group 1 (history of Covid-19) n=88	Group 2 (no history of Covid-19) n=91	*p*-value
**Age (mean±sd)**	32.14±(4.773)	31.42±(4.971)	0.325^1^
**FSH (mean±sd)**	6.394±1.3778	6.043±1.5478	0.111
**BMI (mean±sd)**	26.3 ±3.2	26.4±3.5	0.707
**Antral follicle count (mean±sd)**	5.614±3.1291	6.374±3.7324	0.142
**Infertility type n (%)** Primary Secondary	42 (47.7%) 46 (52.3%)	52 (57.1%) 39 (42.9%)	0.233^2^
**Causes of infertility n (%)** Male Female Both Unexplained	16 (18.2%) 50 (56.8%) 11 (12.5%) 11 (11.5%)	18 (19.8%) 49 (53.8%) 5 (5.5%) 19 (20.9%)	0.209
**Duration of stimulation (mean±sd)**	10.32±1.255	10.13±1.368	0.344
**Number of Oocytes retrieved (mean±sd)**	10.93±6.739	11.89±7.484	0.370
**MII number**	7.92±4.840	8.895.859	0.233
**ET number (mean±sd)**	2.13±.907	2.24±.947	0.401
**Grade 1-2 embryos (mean±sd)**	1.51±1.322	1.43±1.309	0.674
**ET day (mean±sd)**	3.59±.997	3.76±1.013	0.301

Significant p-value at a level of <0.05

1 Student’s t-test was used in the comparison of quantitative variables of parametric data.

2 Chi-squared test was used in the comparison of categorical data.

**Figure 1:**
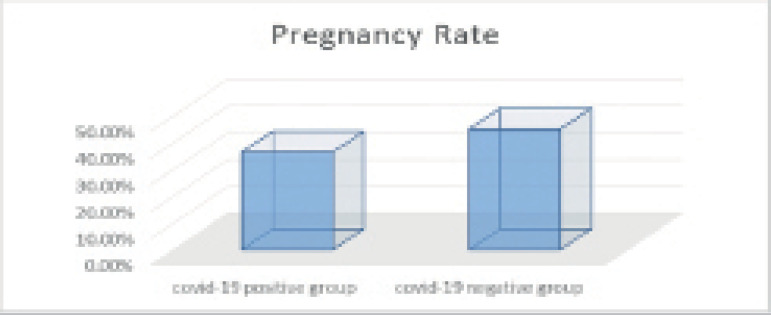
Pregnancy rates in both groups.

**Figure 2:**
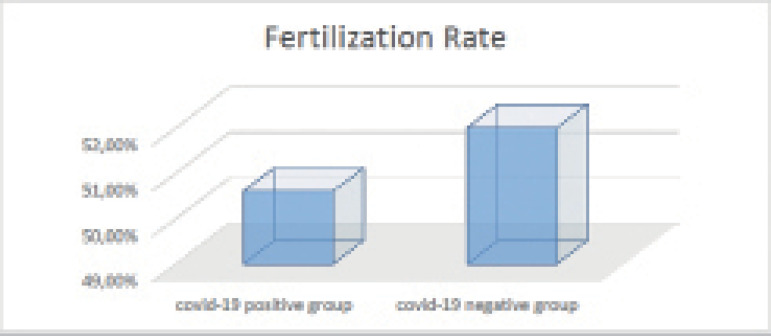
Fertilization rates in both groups.

Regarding clinical pregnancy and fertilization rates, despite the higher pregnancy (45.1% *vs.* 36.4%) and fertilization (52% *vs.* 50.6%) rates seen in Group 2, the differences were not statistically significant (*p*=0.264 for clinical pregnancy rate and *p*=0.647 for fertilization rate) (Table 2, Figures 1 and 2).

**Table 2:** Pregnancy and fertilization rates in both groups.

Variable	Group 1 (n=88)	Group 2 (n=91)	*p*-value
**Pregnancy rate**	32 (36.4%)	41 (45.1%)	0.364
**Fertilization rate**	50.6%	52%	0.647

## DISCUSSION

Almost two years into the COVID-19 pandemic, infertile patients continue to struggle emotionally and mentally. Pandemic fatigue, information overload and misinformation, fear, confusion and other psychological factors are combining to threaten and undermine the pathway back to normalcy (ASRM, 2021).

The novel coronavirus (SARS-CoV-2) pandemic has swept the globe and resulted in significant social and economic disruption, including the largest global recession since the Great Depression of the 1930s. Even though millions of people have been vaccinated, COVID-19 prevention should remain a top priority to reduce the likelihood of the emergence of additional new SARS-CoV-2 variants (WHO, 2021b).

Patients rely on physicians to practice evidence-based medicine supported by facts and scientific data. The ethical and professional responsibility of physicians is to share information publicly to represent current scientific evidence accurately and faithfully. Misrepresentation or misapplication of science is unethical, unprofessional, and harmful to patients and the public.

We do not know how long the current pandemic will last, but it is reasonably certain that SARS-CoV-2 will return cyclically for years, if not decades. Thus, one of the most critical questions that remain to be answered is if or how COVID-19 affects IVF/ICSI cycle outcomes. Hence, we decided to carry out this study.

To our knowledge, this is one of the first studies that investigated the effect of previous exposure to COVID-19 on the outcomes of ICSI cycles.

### Principal Findings

The groups were not significantly different in terms of demographic data. The comparison of the baseline characteristics of both groups revealed that they were not significantly different in terms of duration of stimulation, total dose of stimulation, number of oocytes retrieved, number of metaphase 2 oocytes (MII), number of grade 1-2 embryos, number of transferred embryos, day of embryo transfer, or fertilization rate. Although the fertilization rate was higher in patients without a history of COVID-19 (52% *vs*. 50.6%), the difference is not statistically significant.

The clinical pregnancy rate was also higher in patients without a history of COVID-19 (45.1% *vs*. 36.4%), although not significantly. We do not know the exact reason for these findings, but it may be due to thrombophilic disorders in patients with a history of COVID-19, since the novel coronavirus has proven unusual with respect to the spectrum of its pathological effects (Spratt & Buchsbaum, 2020). Macro- and micro-vascular thrombosis in venous and arterial beds along with venous thromboembolic events (VTEs) occur with a troublesome frequency (Terpos *et al*., 2020).

A recent study found increased platelet activation and aggregation in patients infected with SARS-CoV-2, with increased expression of platelet adhesion protein P-selectin along with altered gene expression in multiple pathways, which may underlie platelet hyper reactivity contributing to thrombo-inflammation in COVID-19 (Manne *et al*., 2020).

In spite of the fact that the underlying mechanisms of COVID-associated hypercoagulability are unclear, multiple laboratory abnormalities related to coagulation occur commonly in hospitalized patients with COVID-19, including increased levels of D-dimer, fibrinogen, fibrin, fibrinogen degradation products, and cytokines, as well as decreased antithrombin, variable platelet counts over the course of disease, and platelet-fibrin micro-thrombi in the pulmonary arterial vasculature on early autopsy studies (Thachil *et al.,* 2020).

When the clinical complications of a disease cannot be explained by known physiology or pathophysiology, designing effective diagnostic or treatment strategies can be extraordinarily difficult. Several issues complicate matters. COVID-19 has a variety of coagulation effects that appear to differ between individuals. Although we are still primarily at an observational stage, with clinicians and clinical researchers learning more about hypercoagulability manifestations of COVID-19, more efforts should be promoted to explore potential interactions between SARS-CoV-2 and pregnancy or estrogen therapy that could guide clinical management (Spratt & Buchsbaum, 2020).

As more information is revealed regarding the effects of SARS-CoV-2 on coagulation, inquiries arise as to whether infection by this virus aggravates the risk of hypercoagulability with pregnancy and estrogen therapy, since this issue has to be considered and may explain why the clinical pregnancy rate was little bit lower in the study group.

### What do we know now?

The novel coronavirus invades target cells by binding to angiotensin converting enzyme 2 (ACE2), which is widely expressed in the ovaries, uterus, vagina and placenta. Significantly, the SARS-CoV-2 is said to interrupt female fertility by regulating ACE2 (Li *et al*., 2021). Accumulating evidence now suggests that SARS-CoV /ACE2 may interfere with female reproductive function, leading to menstrual disorder, infertility, and fetal distress (Jing *et al*., 2020).

ACE2 is a receptor for SARS-CoV (Li *et al*., 2003). The protein expression profile of ACE2 is also considered to be the host receptor of SARS-CoV-2 (Hikmet *et al*., 2020). Thus, SARS-CoV-2 can invade target host cells by using ACE2 as the primary receptor binding site (Li *et al*., 2021), and regulate the expression of ACE2 in host cells (Jing *et al*., 2020). ACE2 expression has been assessed in various human organs, such as respiratory tracts, heart, kidney, ovary, uterus, testis, vagina and placenta, and the gastrointestinal system (Jing *et al.,* 2020; Lippi *et al*., 2020). Notably, ACE2 is highly expressed in the ovaries. Published reports suggest that ACE2 is expressed in stromal cells, granulosa cells and oocytes in immature rat ovaries (Pereira *et al*., 2009). ACE2 regulates follicular development and ovulation, regulates luteal angiogenesis and degeneration, and affects the regular changes of endometrial tissue and embryo development (Jing *et al*., 2020). ACE2 was also expressed in the endometrium, to a greater extent in epithelial than stromal cells. Moreover, the expression of ACE2 changed with the menstrual cycle, being more abundant in the secretory than in the proliferative phase; this could interfere with local Ang-II homeostasis and regulate endometrial regeneration (Vaz-Silva *et al.,* 2009).

Based on recent research, it is possible to suggest ways in which SARS CoV- 2 might affect female fertility: (i) SARS-CoV-2 might attack ovarian tissue and granulosa cells, and decrease ovarian function and oocyte quality, leading to female infertility or miscarriage; and (ii) SARS-CoV-2 might damage endometrial epithelial cells and affect early embryo implantation (Vaz-Silva *et al*., 2009). Considering these factors, SARS-CoV-2 may interrupt female fertility by attacking ovarian tissue and granulosa cells or damaging endometrial epithelial cells (Li *et al*., 2020).

Basigin (BSG) is also one of the most crucial receptors for COVID-19 that mediates its entry into host cells (Mahdian *et al*., 2020). BSG is expressed not only in the uterus, but also in the stroma and granulosa cells of the ovary (Mahdian *et al.,* 2020; Chen *et al*., 2010). BSG may play a role during follicle development, corpus luteum formation, and embryo implantation (Chang *et al*., 2004). Besides, immune system impairment caused by COVID-19 might alter the function of the hypothalamic–pituitary–gonadal axis (Yang *et al*., 2020; Mauvais-Jarvis *et al*., 2020). Sex steroids are potent immune modulators, thus different progesterone and androgen concentrations are likely to influence the immune response and inflammatory outcomes of COVID-19 (Pereira *et al*., 2009). Notwithstanding, the magnitude of the association between COVID-19 and female fertility remains unclear.

### Strengths and limitations

The study showed points of strength and limitations. The power of the study is represented in the strict inclusion and exclusion criteria, which were proven by the non-significant difference regarding baseline characteristics between groups. There is limited data regarding the effects of COVID-19 on the reproductive outcomes of infertile patients undergoing assisted reproduction. To our knowledge, this is one of the first studies that investigated the effects of exposure to COVID-19 and ICSI cycle outcomes.

A major limitation of the current study is its retrospective design and the small sample size. Therefore, the clinical applicability of the study results may be limited until larger randomized clinical trials have been performed.

## CONCLUSION

There is no clear evidence that previous exposure to COVID-19 significantly affects the outcome of ICSI cycles.
